# Editorial: Incisional and Stomal Hernia Prevention

**DOI:** 10.3389/fsurg.2018.00046

**Published:** 2018-07-17

**Authors:** Gabriel Sandblom

**Affiliations:** Department of Surgery, Karolinska Institutet, Södersjukhuset, Solna, Sweden

**Keywords:** incisional hernia, mesh augmentation, abdominal wall closure, open abdomen, stomal hernia

Ever since abdominal wall surgery became routine, the occurrence of incisional or stomal hernia has been considered an inevitable late complication of any abdominal procedure. Large incisional hernia used to be considered a complicated condition with a high recurrence rate following attempted repair. Even though the introduction of mesh techniques and the endoscopic approach has made it possible to repair incisional hernia with greater success and with much less risk than two decades ago, incisional and stomal hernias continue to be a public health problem, causing disability and poor quality of life for the patient, and a great cost to society.

Recent studies, however, have shown that most incisional hernias may be avoided by using an adequate technique. The questions: how much effort, cost, and risk for other side-effects can we accept to prevent this condition? Recently published guidelines on midline incision closure recommend using slowly resorbable continuous sutures with small bites (Fortelny). The use of small bites at short intervals may reduce the risk for incisional hernia by one half compared to closure with large bites. Although this requires patience and time at the end of an abdominal procedure, a stage when focus on surgical excellence is no longer considered a major issue, the extra minutes spent on the quality of closure to avoid incisional hernia may lead to a substantial increase in health-related quality-of-life for the patient, and avoid further surgical procedures. This is of special concern when closing the wound after open abdomen treatment (Berrevoet).

The risk for incisional hernia may be reduced by almost 90% by reinforcing the suture line with mesh (Fortelny). Over the last 5 years, a very large number of reports on the effectiveness of mesh reinforcement have been presented, all of them showing that the risk for incisional hernia may be virtually eliminated in selected patient groups (Argudo et al.). However, since the use of mesh is a preventive measure, and it is difficult to select those patients likely to benefit from its use, the level of acceptance of potential side-effects is low. Side-effects do occur with mesh, and the decision to use mesh for suture line reinforcement should thus be taken with care. Furthermore, more research is required into the optimal anatomical space for placement of the mesh, and which kind of mesh is most suitable for this purpose.

After stomal closure, a defect is left in the abdominal wall. This dilemma has traditionally been solved by suturing the external oblique aponeurosis. Several studies, however, have shown that the risk for incisional hernia through the stomal opening is very high, probably due to the tension in the tissue caused by the aponeurosis defect. Research into the application of preventive mesh during closure is therefore necessary (Harries and Torkington).

Prophylactic mesh may also be used to prevent stomal hernia; though the results of studies to date are not as convincing as with incisional hernia prevention. Most studies on stomal hernia prevention have been carried out in open surgery, and there are few studies on stomal hernia prevention in laparoscopic surgery (López-Cano and Rodriguez). Laparoscopic surgery is widely accepted as an approach that minimizes trauma to the abdominal wall. Nevertheless, the stomal opening in laparoscopic surgery is still a weak point in the abdominal wall.

The increasing interest in prevention rather than treatment of manifest incisional and stomal hernias may lead to a greater increase in the cost-effectiveness of abdominal surgery, a reduction in morbidity, and better health-related quality-of-life. Development of incisional and stomal hernia, however, must be taken into consideration when assessing the outcome of an abdominal procedure if we are to make future progress. Late abdominal wall complications tend to be neglected when evaluating the safety of a surgical intervention since they often take several years to develop. Nevertheless, when performing surgery, late complications must be considered, as well as the benefits and potential risks of all measures taken to prevent them when closing the abdominal wall. More studies are required to further develop techniques used for suture line reinforcement.

## Author contributions

The author confirms being the sole contributor of this work and approved it for publication.

### Conflict of interest statement

The author is a scientific advisor of Novus Scientific.

